# Computer vision syndrome among medical students at Kasr Al-Ainy Faculty of Medicine, Cairo University, Egypt: a cross-sectional study of prevalence, risk factors, and protective measures

**DOI:** 10.3389/fpubh.2026.1809944

**Published:** 2026-05-07

**Authors:** Mostafa A. Khalifa, Mostafa Atef Diab, Mohamed Ramadan, Ibraheem AbuAlrub, Shehabeldin S. Anan

**Affiliations:** 1 Faculty of Medicine, Cairo University, Cairo, Egypt; 2 Faculty of Medicine, Suez University, Suez, Egypt; 3 Department of Clinical Medical Sciences, Faculty of Medicine, Palestine Polytechnic University, Hebron, Palestine

**Keywords:** digital eye strain, eye health, medical students, public health, screen time

## Abstract

**Background:**

Computer vision syndrome (CVS), also known as digital eye strain, refers to a group of eye- and vision-related problems resulting from prolonged use of digital screens. With the increasing dependence on digital devices for learning, especially among university students, CVS has emerged as a growing public health concern. Medical students are particularly at risk due to extended screen time for academic purposes.

**Objective:**

This cross-sectional study aimed to assess the prevalence, risk factors, and protective measures associated with CVS among medical students at Kasr Al-Ainy Faculty of Medicine, Cairo University.

**Methods:**

A cross-sectional study was conducted using a self-administered electronic questionnaire based on the validated Computer Vision Syndrome Questionnaire (CVS-Q). The survey was distributed to 1,986 participants, with 1,882 responses included for analysis after exclusions.

**Results:**

The results revealed a high prevalence of CVS (72.4%), with symptoms such as eye strain, dryness, and headaches being most common. Female sex, lower academic levels, pre-existing eye conditions, smoking, and prolonged screen time (especially on tablets or iPads) were identified as significant risk factors. Protective measures, including screen brightness adjustment, frequent breaks, and consulting eye specialists, were associated with reduced CVS symptoms.

**Conclusion:**

CVS is highly prevalent among medical students and significantly associated with modifiable risk factors, particularly prolonged screen time and poor screen ergonomics. Targeted interventions, including optimizing screen settings, limiting continuous use, and taking regular breaks, may reduce the symptom burden.

## Introduction

Computer vision syndrome (CVS), also referred to as digital eye strain, comprises a spectrum of visual and ocular symptoms arising from extended use of electronic devices such as computers, tablets, and smartphones. These symptoms include eye strain, dryness, blurred vision, and headaches, which are increasingly prevalent in today’s digital age (1).

The COVID-19 pandemic increased reliance on digital devices for work, education, and social interaction, leading to longer screen time without sufficient breaks or ergonomic practices, thereby raising concerns about CVS (2).

A systematic review reported that CVS prevalence rose to approximately 74% during the COVID-19 pandemic, highlighting the need for awareness and preventive strategies in the current digital landscape (3).

CVS exhibits varying prevalence rates across different populations, influenced by factors such as screen time duration, posture, and awareness of preventive measures. Among medical students, a study at the University of Khartoum reported a 94% prevalence of CVS symptoms, with 72.4% experiencing at least three symptoms, highlighting the impact of prolonged device usage and poor posture (4). Similarly, undergraduate students at the National University of Malaysia exhibited a 63% prevalence, with significant associations found between CVS and factors such as refractive errors, improper screen distance, and poor posture (5). Radiologists, due to their extensive screen time, are also at risk; a national survey in Saudi Arabia reported a 65.4% prevalence among radiologists, with symptoms such as headaches, dryness, and blurred vision being common (6). Children have not been spared, especially during the COVID-19 pandemic; a study in India found a significant prevalence of digital eye strain among children engaged in online learning, emphasizing the need for awareness and preventive measures in this age group (7).

Medical students have experienced increased screen time due to the transition to online learning, making them more susceptible to CVS. A study conducted at the University of Illinois at Chicago reported a CVS prevalence of 69.1% among medical students, highlighting the impact of remote education on ocular health (8).

Given the high prevalence of CVS among medical students and its potential impact on academic performance and quality of life, it is imperative to assess the associated risk factors and develop effective strategies for prevention and management (9).

Despite the high prevalence, there remains a scarcity of comprehensive studies addressing this issue in the region. For instance, a study conducted at the University of Khartoum reported a 94% prevalence of CVS symptoms among medical students (4). Similarly, research at King Abdulaziz University in Jeddah found that 95% of undergraduate medical students reported at least one symptom of CVS (10). In Egypt, a study at Sohag University Hospital indicated that 86% of medical students experienced one or more CVS manifestations (11). However, these studies often had limited sample sizes and did not extensively assess associated risk factors.

The selection of risk factors in this study was guided by a conceptual framework informed by previous literature, categorizing the contributors to computer vision syndrome into behavioral factors (e.g., screen time, device use, and break patterns), environmental factors (e.g., viewing distance and screen brightness), and visual-related factors (e.g., use of corrective lenses and ocular symptoms).

Our study aimed to address this gap by utilizing a large sample size (*n* = 1,882), employing a validated assessment tool (Computer Vision Syndrome Questionnaire (CVS-Q)), and providing a detailed evaluation of multiple behavioral, environmental, and visual-related risk factors. In addition, it represents one of the largest studies conducted among medical students in Egypt and was carried out at Kasr Al-Ainy Faculty of Medicine, Cairo University, one of the largest and most established medical schools in the Middle East, thereby providing a more detailed understanding of CVS among medical students in the region.

## Methodology

A cross-sectional descriptive study was conducted between March and May 2025 among medical students enrolled at Kasr Al-Ainy Faculty of Medicine to assess the prevalence of CVS. The sample size was determined using the Raosoft sample size calculator (12), with *α* = 0.05, a 95% confidence interval, an estimated population size of 100,000, and an estimated prevalence of 50% due to the lack of prior information on the prevalence of CVS among students. The minimum required sample size was 383; however, 1,882 responses were collected, which exceeded the minimum requirement. A total of 1,986 students were initially surveyed using a self-administered electronic questionnaire adapted from previously validated surveys. Individuals were invited to participate via social media platforms, email, and face-to-face recruitment on the university campus using convenience sampling methods, taking into consideration a representative distribution across academic levels and sex. Among them, 104 were excluded due to a history of systemic diseases affecting vision, leaving a final sample of 1,882 students for analysis, which yielded a response rate of 1,882/1,986*100% = 95%.

The questionnaire covered demographics, daily screen time, types of devices used, viewing habits, preventive measures, and the impact of dry eye syndrome (DES) symptoms on daily life, including academic performance, social interactions, and personal well-being. Symptoms such as eye strain, dryness, redness, blurred vision, and headaches were assessed for frequency and intensity over the past week and scored to classify the severity of DES. The diagnosis of Computer Vision Syndrome (CVS) was based on the Computer Vision Syndrome Questionnaire (CVS-Q), as validated by Alfarhan et al. The CVS-Q assesses both the frequency and intensity of 16 ocular and visual symptoms. Frequency was scored as follows: never = 0, occasionally = 1, and often/always = 2; meanwhile, intensity was scored as moderate = 1 and intense = 2. A composite score for each symptom was calculated by multiplying frequency by intensity. The total CVS score was obtained by summing all symptom scores. Participants were classified as having CVS if their total CVS-Q score was ≥6, in accordance with the original scoring system of the CVS-Q. Severity of CVS was classified as mild if the CVS score was between 6 and 12, moderate if the CVS score was between 13 and 18, and severe if the CVS score was 19 or more. Students were also asked about their awareness of the 20–20-20 rule and other preventive behaviors, such as using blue light filters or taking regular breaks.

Participants were included if they used digital screens for at least 2 h daily and had 6/6 vision (corrected or uncorrected). Those with active systemic conditions affecting vision, a history of recent ocular surgery, or the use of vision-altering medications were excluded.

All data were entered into Statistical Product and Service Solutions (SPSS) for Windows, version 26, and were used for descriptive and inferential analysis. Descriptive statistics for continuous variables were presented using means and standard deviations, whereas categorical variables were summarized using frequencies and percentages. The mean differences were assessed using the independent samples *t*-test and analysis of variance (ANOVA), while the Pearson’s chi-squared test was used for categorical variables. Binary logistic regression was performed, and all variables with a *p* **<** 0.2 were included in the regression model. All tests were two-tailed, and a *p* ≤ 0.05 was considered statistically significant.

## Results

### Demographic characteristics

A total of 1,986 participants were initially included in the study via an online-based questionnaire. Among them, 104 participants were excluded due to systemic diseases affecting vision, leaving 1,882 participants who took part in the study (response rate = 1,882/1,986*100% = 95%). The mean age was 21.3 ± 2.44 years. The sample consisted primarily of female individuals (62.4%) and students who were unmarried (98.2%). The majority of participants resided in urban areas (87.5%).

The study reported that 92% of participants were non-smokers, 4.9% were current smokers, and 3.1% were former smokers. Traditional cigarettes and e-cigarettes were the most commonly used tobacco products, reported by 84 (56%) and 56 (37%) of smokers, respectively. In addition, 46.2% of participants did not require glasses, while eye conditions were reported by 19.3% of the surveyed students (Table 1).

**Table 1:** Demographic characteristics of the study sample (*n* = 1,882).

Demographic characteristics	Frequency (%)
Age (mean ± SD)	21.3 ± 2.44
Sex	
Male	708(37.6)
Female	1,174(62.4)
Study level	
1st year	343(18.2)
2nd year	282(15)
3rd year	240(12.8)
4th year	290(15.4)
5th year	242(12.9)
6th year	311(16.5)
7th year	174(9.2)
Smoking status	
Non-smoker	1,732(92)
Current smoker	92(4.9)
Former smoker	58(3.1)
Types of smoking (among the 150 participants who have used tobacco products)	
Traditional cigarettes	84(56)
Waterpipe	56(37)
E-cigarettes	87(58)
Marital status	
Single	1849(98.2)
Married	33(1.8)
Place of living	
Rural	235(12.5)
Urban	1,647(87.5)
Wearing glasses	
No	869(46.2)
Yes, but vision is not completely corrected	443(23.5)
Yes, and vision is completely corrected	570(30.3)
Suffering from any eye condition (Yes)	364(19.3)

### Screen-related habits

A majority (65.7%) of participants used screens for more than 6 h daily. A total of 88.3% relied on smartphones as their primary device, and 32.9% used either tablets/iPads or laptops, according to survey results. During their study time, students spent substantial portions of time watching media, entertaining themselves on social networks, and studying (80.3 and 80.4 and 74.5%, respectively). Among the participants, 42.9% reported holding their screen at a distance of less than 30 cm. The majority of frequent screen users reported setting their brightness levels to maximum (53.1%), while 31.3% rarely took screen breaks. The most commonly reported break duration was 5–10 min, since 30.4% of participants followed this pattern (Table 2).

**Table 2:** Screen-related habits of the study participants (*n* = 1,882).

Habits	Frequency (%)
Hours of daily screen use	
Less than 2 h	11(0.6)
2–4 h	142(7.5)
4–6 h	491(26.1)
6–8 h	618(32.8)
More than 8 h	620(32.9)
Type of screens used **	
Smartphone	1,661(88.3)
Laptop	542(28.8)
Tablet or iPad	619(32.9)
Desktop	91(4.8)
Main reasons for using digital screens **	
Social media	1,511(80.3)
Studying/online classes	1,439(74.5)
Entertainment	1,514(80.4)
Gaming	277(14.7)
Working	35(1.9)
Distance from the screen	
Less than 30 cm	807(42.9)
30–50 cm	1,025(54.5)
More than 50 cm	50(2.7)
Screen brightness	
Dull	796(42.3)
Bright	999(53.1)
Very bright	87(4.6)
Break frequency	
Rarely	590(31.3)
Every 30 min	429(22.8)
Every 1 h	566(30.1)
Every 1–2 h	297(15.8)
Break duration	
Less than 5 min	490(26)
5–10 min	573(30.4)
10–20 min	349(18.5)
More than 20 min	470(25)

**Participants could choose more than one response, so the total percentage could be higher than 100%.

### Eye symptoms and computer vision syndrome

The study diagnosed Computer Vision Syndrome (CVS) in 72.4% of participants. Students frequently experienced symptoms of burning eyes, with 48.6% reporting occasional occurrences and 10.8% reporting frequent occurrences. Eye redness was reported by 36.4% of participants as occasional and by 8.9% as frequent. This was followed by dryness symptoms, with 34.4% reporting occasional occurrences and 17.7% reporting frequent occurrences. Approximately 28.4% of participants experienced light sensitivity, and a similar percentage reported blurred vision. Headaches were reported by 32.3% of participants. In terms of severity, 52.9% of participants experienced mild CVS, 23.2% had moderate symptoms, and 23.9% experienced severe symptoms. Further details regarding these symptoms and their intensity are presented in Table 3.

**Table 3:** Distribution of eye symptoms by intensity and frequency among the study participants (*n* = 1,882).

Symptoms	Frequency			Intensity	
	Never	Occasionally	Often	Moderate	Intense
1. Burning eyes	764(40.6)	915(48.6)	203(10.8)	956(85.5)	162(14.5)
2. Itching eyes	887(47.1)	817(43.5)	178(9.5)	854(85.8)	141(14.2)
3. Feeling of a foreign body in the eyes	1,058 (56.2)	685(36.4)	139(7.4)	712(86.4)	112(13.6)
4. Tearing eyes	784(41.7)	871(46.3)	227(12.1)	917(83.5)	181(16.5)
5. Excessive blinking	1,145 (60.8)	584(31)	153(8.1)	613(83.2)	124(16.8)
6. Eye redness	1,030 (54.7)	685(36.4)	167(8.9)	687(80.6)	165(19.4)
7. Eye pain	936(49.7)	763(40.5)	183(9.7)	748(79.1)	198(20.9)
8. Heavy eyelids	1,211(64.3)	514(27.3)	157(8.3)	532(79.3)	139(20.7)
9. Dryness in eyes	901(47.9)	647(34.4)	334(17.7)	672(68.5)	309(31.5)
10. Blurred vision	1,029(54.7)	654(34.8)	199(10.6)	669(78.4)	184(21.6)
11. Double vision	1,597(84.9)	225(12)	60(3.2)	231(81.1)	54(18.9)
12. Difficulty focusing for near vision	1,273(67.6)	489(26)	120(6.4)	483(79.3)	126(20.7)
13. Increased sensitivity to light	963(51.2)	667(35.4)	252(13.4)	658(71.6)	261(28.4)
14. Colored halos around objects	1,448(76.9)	345(18.3)	89(4.7)	361(83.2)	73(16.8)
15. Feeling that sight is worsening	1,052(55.9)	597(13.7)	233(12.4)	633(76.3)	197(23.7)
16. Headache	475(25.2)	935(49.7)	472(25.1)	953(67.7)	454(32.3)
CVS score (mean ± SD)			11.33 ± 9.05		
Computer vision syndrome (Yes)			1,362(72.4)		
Computer vision syndrome severity					
Mild			721(52.9)		
Moderate			316(23.2)		
Severe			325(23.9)		

### Eye protection behaviors and awareness

The majority of participants were aware of the major effects of digital exposure through mobile screens, as 55.4% recognized digital eye strain or CVS and 27.4% understood the 20–20-20 rule. In terms of preventive practices, the majority of students reported using three types of protective measures, including reducing screen brightness (51.5%), applying screen filters (49.5%), and wearing blue light-blocking glasses (29.2%). A significant proportion of participants (31.7%) sought consultation with eye care specialists primarily due to screen-related problems. Eye symptoms caused mild disruption to daily routines in 45% of respondents, while severe disruption was reported by 4% of participants (Table 4).

**Table 4:** Eye protection behaviors and awareness of eye–screen interaction among the study participants (*n* = 1,882).

Behavior	Frequency (percentage)
Awareness of digital eye strain or computer vision syndrome (*Yes*)	1,043(55.4)
Using the following protective measures	
Blue light-blocking glasses	549(29.2)
Screen brightness adjustment	970(51.5)
Screen filters (e.g., night mode, dark mode)	931(49.5)
Lubricating eye drops	436(23.2)
Taking frequent breaks	530(28.2)
Awareness of the 20–20-20 rule (Yes)	515(27.4)
Consultation with an eye specialist due to screen-related eye issues (Yes)	596(31.7)
Eye symptoms interfering with daily activities	
No impact	626(33.3)
Mild impact	846(45)
Moderate impact	334(17.7)
Severe impact	76(4)

### Associations with CVS and its severity

The data showed that CVS was more prevalent among female individuals, students with lower levels of medical education, and participants who wore glasses (*p* < 0.001). Participants with pre-existing eye diseases had a significantly higher likelihood of developing CVS (*p* < 0.001). In contrast, using screen brightness adjustment as a protective measure and consulting an eye specialist were associated with a reduced prevalence of CVS (*p* < 0.001) (Table 5).

**Table 5:** Association between different demographic variables, behavioral factors, and diagnosis of Computer vision syndrome using the chi-squared test among the study participants (*n* = 1,882).

Demographic variable	Subgroups	Computer vision syndrome		*P*-value
		Yes	No	
Age (mean ± SD)		21.2 ± 2.5	21.7 ± 2.3	>0.001
Sex	Male	437(61.7)	271(38.3)	>0.001
	Female	925(78.8)	249(21.2)	
Study level	1st year	270(78.7)	73(21.3)	>0.001
	2nd year	221(78.4)	61(21.6)	
	3rd year	181(75.4)	59(24.6)	
	4th year	194(66.9)	96(33.1)	
	5th year	176(72.7)	66(27.3)	
	6th year	203(65.3)	108(34.7)	
	7th year	117(67.2)	57(32.8)	
Smoking status	Non-smoker	1,256(72.5)	476(27.5)	0.888
	Current smoker	65(70.7)	27(29.3)	
	Former smoker	41(70.7)	17(29.3)	
Traditional cigarettes	Yes	59(70.2)	25(29.8)	0.897
	No	47(71.2)	19(28.8)	
Waterpipe	Yes	38(67.9)	18(32.1)	0.560
	No	68(72.3)	26(27.7)	
E-cigarettes	Yes	60(69)	27(31)	0.591
	No	46(73)	17(27)	
Marital status	Single	1,339(72.4)	510(27.6)	0.729
	Married	23(69.7)	10(30.3)	
Place of living	Rural	180(76.6)	55(23.4)	0.121
	Urban	1,182(71.8)	465(28.2)	
Wearing glasses	No	573(65.9)	296(34.1)	>0.001
	Yes, but vision is not completely corrected	368(83.1)	75(16.9)	
	Yes, and vision is completely corrected	421(73.9)	149(26.1)	
Pre-existing eye condition	Yes	326(89.6)	38(10.4)	>0.001
	No	1,036(68.2)	482(31.8)	
Hours of daily use	Less than 2 h	3(27.3)	8(72.7)	>0.001
	2–4 h	87(61.3)	55(38.7)	
	4–6 h	340(69.2)	151(30.8)	
	6–8 h	545(73.5)	164(26.5)	
	More than 8 h	478(77.1)	142(22.9)	
Smartphone use	Yes	1,199(72.2)	462(27.8)	0.642
	No	163(73.8)	58(26.2)	
Laptop use	Yes	387(71.4)	155(28.6)	0.550
	No	975(72.8)	365(27.2)	
Tablet or iPad use	Yes	485(78.4)	134(21.6)	>0.001
	No	877(69.4)	386(30.6)	
Desktop use	Yes	63(69.2)	28(30.8)	0.492
	No	1,299(72.5)	492(27.5)	
Social media as a reason for screen use	Yes	1,087(71.9)	424(28.1)	0.399
	No	275(74.1)	96(25.9)	
Studying/online classes as a reason for screen use	Yes	1,057(73.5)	382(26.5)	0.058
	No	305(68.8)	138(31.2)	
Entertainment as a reason for screen use	Yes	1,089(71.9)	425(28.1)	0.385
	No	273(74.2)	95(25.8)	
Gaming as a reason for screen use	Yes	197(71.1)	80(28.9)	0.614
	No	1,165(72.6)	440(27.4)	
Working as a reason for screen use	Yes	28(80)	7(20)	0.308
	No	1,334(72.2)	513(27.8)	
Distance from the screen	Less than 30 cm	594(73.6)	213(26.4)	0.499
	30–50 cm	734(71.6)	29.1(28.4)	
	More than 50 cm	34(68.0)	16(32)	
Screen brightness	Dull	574(72.1)	222(27.9)	0.223
	Bright	718(71.9)	281(28.1)	
	Very bright	70(80.5)	17(19.5)	
Break frequency	Every 30 min	289(67.4)	140(32.6)	0.050
	Every 1 h	412(72.8)	154(27.2)	
	Every 1–2 h	225(75.8)	72(24.2)	
	Rarely	436(73.9)	154(26.1)	
Break duration	Less than 5 min	359(73.3)	131(26.7)	0.890
	5–10 min	415(72.4)	158(27.6)	
	10–20 min	254(72.8)	95(27.2)	
	More than 20 min	334(71.1)	136(29.9)	
Awareness of digital eye strain or computer vision syndrome	Yes	757(72.6)	286(27.4)	0.821
	No	605(72.1)	234(27.9)	
Blue light-blocking glasses as a protective measure	Yes	414(75.4%)	135(24.6%)	0.058
	No	948(71.1%)	385(29.9%)	
Screen brightness adjustment as a protective measure	Yes	682(70.3)	288(29.7)	0.039
	No	680(74.6)	232(25.4)	
Screen filters as a protective measure	Yes	693(74.4)	238(25.6)	0.047
	No	669(70.3)	282(29.7)	
Lubricating eye drops as a protective measure	Yes	389(89.2)	47(10.8)	>0.001
	No	973(67.3)	473(32.7)	
Taking frequent breaks as a protective measure	Yes	384(72.5)	146(27.5)	0.960
	No	978(72.3)	374(27.7)	
Awareness of the 20–20-20 rule	Yes	376(73)	139(27)	0.703
	No	986(72.1)	381(27.9)	
Consultation with an eye specialist due to screen-related eye issues?	Yes	506(84.9)	90(15.1)	>0.001
	No	856(66.6)	430(33.4)	
Eye symptoms interfering with daily activities	No impact	295(47.1)	331(52.9)	>0.001
	Mild impact	698(82.5)	148(17.5)	
	Moderate impact	302(90.4)	32(9.6)	
	Severe impact	67(88.2)	9(11.8)	

Sex (*p* < 0.001), smoking status (*p* = 0.023), and wearing glasses (*p* < 0.001) were identified as significant risk factors for CVS severity. Higher severity levels of CVS were observed among female students. Severe CVS was more prevalent among smokers (35.4%) compared to non-smokers (23.5%). Individuals with incomplete vision correction showed the highest proportion of severe CVS (30.4%). Increasing daily screen use hours, studying on screens, and tablet or iPad use were associated with a higher prevalence of severe CVS symptoms. In contrast, taking frequent breaks and consulting an eye specialist were associated with reduced severity of CVS symptoms (*p* < 0.05) (Table 5).

## Discussion

Our study findings can be categorized into three main groups. The first includes factors associated with significantly higher CVS prevalence, such as female sex, lower academic level, wearing glasses, pre-existing eye disease, smoking, and incomplete vision correction. The second comprises behavioral factors influencing CVS prevalence, including longer daily screen time and studying on screens, particularly tablets or iPads. The third includes protective factors associated with lower CVS prevalence, such as adjusting screen brightness, taking frequent breaks, and consulting an eye specialist.

CVS diagnosis was based on the validated CVS-Q. A study by Haya assessed the questionnaire’s validity, unidimensionality, and differential item functioning. The study concluded that the scale can be confidently used by academic and industry professionals, as it provides accurate and reliable CVS -Q frequency items (13).

Regarding factors associated with CVS prevalence, our study found a significantly higher prevalence of CVS among female individuals, although some studies reported that the difference was not statistically significant (14, 15). Cantó-Sancho et al. attributed the male predominance reported by Larese et al. to the latter’s focus on visual fatigue as an isolated symptom rather than considering CVS as a broader condition encompassing multiple ocular surface symptoms, including dryness (16). Moreover, they assessed the frequency of eye symptoms associated with digital device use without specifying the instrument employed or clarifying whether a validated tool was used. Moreover, it is also noteworthy that a higher male-to-female ratio was reported among CVS groups, which is clinically relevant (14, 17–20). The sex-related differences may be explained by the influence of sex hormones such as androgens and estrogens, along with other hormonal factors, including hypothalamic–pituitary hormones, glucocorticoids, insulin, insulin-like growth factor 1, and thyroid hormones, as well as differences in sex chromosome composition (21).

Our study found that low levels of education were significantly associated with CVS prevalence. This finding aligns with the study by Wang et al., who found the prevalence to be higher in undergraduate students than in medical students (8). Similarly, the study by Nagwa et al. showed a higher prevalence in the earlier years of medical education (22). One of the factors contributing to this finding is that students in the early years of medical education often have a greater volume of online classes (23).

Our study showed a higher prevalence of CVS among participants with underlying eye disease and those wearing glasses. In their study, Wang et al. reported that wearing glasses is associated with a significantly higher prevalence of CVS, which aligns with our findings (8). This can be attributed to multiple factors, including uncorrected errors of refraction, increased accommodative demand, and inadequate optical correction for screen use (24–26).

Our results showed a significant association between smoking and CVS prevalence. This exact association is believed to be indirect; smoking affects the tear film, putting individuals at a higher risk of dry eyes and further increasing their susceptibility to CVS (13). In addition, oxidative stress that emerges from smoking and smoking-induced vasoconstriction are thought to be contributing factors (27, 28).

Regarding factors that influence CVS prevalence, our results align with previous studies that have highlighted a significant association between longer daily screen time and CVS prevalence (14, 17, 29, 30).

The association between longer daily screen time and CVS prevalence is not confined to medical students. Al Dandan et al. concluded that radiologists, including those who spend 7–9 h per day reviewing medical images, have a higher prevalence of CVS (6). Similarly, Mohan et al. reported that the use of digital devices for more than 5 h in children was associated with a higher prevalence of digital eye strain. Their study also showed that the most commonly reported symptom was headache, which aligns with our findings. This is not the case for the second most common symptom, which was itching in their study and tearing in ours (7).

Our study found that the device associated with the highest prevalence was a tablet or iPad, followed by smartphones. Ibrahim et al. discussed how close viewing distances, poor ergonomic angles, and prolonged usage, especially with handheld devices, can increase CVS symptoms more than desktop use. However, their study found smartphones to be associated with the highest prevalence of CVS (10).

Regarding protective factors against CVS, our study found that adjusting screen brightness plays a significant role in reducing the prevalence of CVS. A previous study showed that watching screens in the dark is associated with higher CVS prevalence (6). Screen glare is also associated with higher CVS (31). Adjusting screen brightness to match ambient lighting may minimize eye strain. This practice helps in reducing glare and prevents the eyes from working harder to adapt to varying light conditions (32).

A study among medical students found a significant association between taking breaks during computer use and relief from CVS symptoms. Students who took regular breaks experienced fewer symptoms such as headaches and dry eyes (33). Another study among Thai university students during virtual classrooms found that inadequate break time between classes was significantly associated with a higher prevalence of CVS. Students who did not take sufficient breaks experienced more severe symptoms (34). This suggests that incorporating regular breaks during screen use is a practical and effective measure to prevent and alleviate CVS symptoms among students and children (Tables 6, 7).

**Table 6:** Association between different demographic variables, behavioral factors, and the severity of computer vision syndrome among the study participants who were diagnosed with computer vision syndrome (*n* = 1,362).

Demographic variable	Subgroups	Computer vision syndrome severity			*P*-value
		Mild	Moderate	Severe	
Age (mean ± SD)		21.3 ± 2.3	21.3 ± 2.9	21 ± 2.4	0.142
Sex	Male	263(60.2)	94(21.5)	80(18.3)	>0.001
	Female	458(49.5)	222(24)	245(26.5)	
Study level	1st year	139(51.5)	59(21.9)	72(26.7)	0.455
	2nd year	105(47.5)	57(25.8)	59(26.7)	
	3rd year	86(47.5)	49(27.1)	46(25.4)	
	4th year	107(55.2)	46(23.7)	41(21.1)	
	5th year	101(57.4)	38(21.6)	37(21)	
	6th	115(56.7)	40(19.7)	48(23.6)	
	7^th^	68(58.0.1)	27(23.1)	22(18.8)	
Smoking status	Non-smoker	669(53.3)	292(23.2)	295(23.5)	0.023
	Current smoker	24(36.9)	18(27.7)	23(35.4)	
	Former smoker	28(68.3)	6(14.6)	7(17.1)	
Traditional cigarettes	Yes	24(40.7)	15(25.4)	20(33.9)	0.147
	No	28(59.6)	9(19.1)	10(21.3)	
Waterpipe	Yes	16(42.1)	11(28.9)	11(28.9)	0.442
	No	36(52.9)	13(19.1)	19(27.9)	
E-cigarettes	Yes	28(46.7)	13(21.7)	19(31.7)	0.680
	No	24(52.2)	11(23.9)	11(23.9)	
Marital status	Single	709(52.9)	310(23.2)	320(23.9)	0.937
	Married	12(52.2)	6(26.1)	5(21.7)	
Place of living	Rural	95(52.8)	48(26.7)	37(20.6)	0.363
	Urban	626(53)	268(22.7)	288(24.4)	
Wearing glasses	No	334(58.3)	124(21.6)	115(20.1)	>0.001
	Yes, but vision is not completely corrected	158(42.9)	98(26.6)	112(30.4)	
	Yes, and vision is completely corrected	229(54.4)	94(22.3)	98(23.3)	
Pre-existing eye condition	Yes	131(40.2)	75(23)	120(36.8)	>0.001
	No	590(56.9)	241(23.3)	205(19.8)	
Hours of daily use	Less than 2 h	0(0)	2(66.6)	1(33.3)	>0.001
	2–4 h	47(54)	19(21.8)	21(24.1)	
	4–6 h	206(60.6)	71(20.9)	63(18.5)	
	6–8 h	257(56.6)	104(22.9)	93(20.5)	
	More than 8 h	211(44.1)	120(25.1)	147(30.8)	
Smartphone use	Yes	644(53.7)	279(23.3)	276(23)	0.126
	No	77(47.2)	37(22.7)	49(30.1)	
Laptop use	Yes	197(50.9)	82(21.2)	108(27.9)	0.079
	No	524(53.7)	234(24)	217(22.3)	
Tablet or iPad use	Yes	210(43.3)	131(27)	144(27.9)	>0.001
	No	511(58.3)	185(21.1)	181(20.6)	
Desktop use	Yes	31(49.2)	18(28.6)	14(22.2)	0.586
	No	690(53.1)	298(22.9)	311(23.9)	
Social media as a reason for screen use	Yes	579(53.3)	250(23)	258(23.7)	0.886
	No	142(51.6)	66(24)	67(24.4)	
Studying/online classes as a reason for screen use	Yes	531(50.2)	253(23.9)	273(25.8)	>0.001
	No	190(62.3)	63(20.7)	52(17)	
Entertainment as a reason for screen use	Yes	580(53.3)	251(23)	258(23.7)	0.892
	No	141(51.6)	65(23.8)	67(24.5)	
Gaming as a reason for screen use	Yes	101(51.3)	54(27.4)	42(21.3)	0.286
	No	620(53.2)	262(22.5)	283(24.3)	
Working as a reason for screen use	Yes	10(35.7)	9(32.1)	9(32.1)	0.182
	No	711(53.3)	307(23)	316(23.7)	
Distance from the screen	Less than 30 cm	292(49.2)	138(23.2)	164(27.6)	0.050
	30–50 cm	410(55.9)	169(23)	155(21.1)	
	More than 50 cm	19(55.9)	9(26.5)	6(17.6)	
Screen brightness	Dull	298(21.9)	136(23.7)	140(24.4)	0.088
	Bright	396(55.2)	161(22.4)	161(22.4)	
	Very bright	27(38.6)	19(27.1)	24(34.3)	
Break frequency	Every 30 min	177(61.2)	57(19.7)	55(19)	>0.001
	Every 1 h	224(54.4)	99(24)	89(21.6)	
	Every 1–2 h	122(45.2)	60(26.7)	43(19.1)	
	Rarely	198(45.4)	100(22.9)	138(31.7)	
Break duration	Less than 5 min	182(50.7)	80(22.3)	97(27)	0.653
	5–10 min	226(54.5)	99(23.9)	90(21.7)	
	10–20 min	129(50.8)	62(24.4)	63(24.8)	
	More than 20 min	184(55.1)	75(22.5)	75(22.5)	
Awareness of digital eye strain or computer vision syndrome	Yes	400(52.8)	172(22.7)	185(24.4)	0.815
	No	321(53.1)	144(23.8)	140(23.1)	
Blue light-blocking glasses as a protective measure	Yes	198(47.8)	98(23.7)	118(28.5)	0.015
	No	523(55.2)	218(23)	207(21.8)	
Screen brightness adjustment as a protective measure	Yes	357(52.3)	165(24.2)	160(23.5)	0.683
	No	364(53.5)	151(22.2)	165(24.3)	
Screen filters as a protective measure	Yes	355(51.2)	160(23.1)	178(25.7)	0.252
	No	366(54.7)	156(23.3)	147(22)	
Lubricating eye drops as a protective measure	Yes	157(40.4)	105(26.2)	130(33.4)	>0.001
	No	564(58)	214(22)	195(20)	
Taking frequent breaks as a protective measure	Yes	216(56.3)	83(21.6)	85(22.1)	0.308
	No	505(51.6)	233(23.8)	240(24.5)	
Awareness of the 20–20-20 rule	Yes	205(54.5)	88(23.4)	83(22.1)	0.822
	No	516(52.3)	228(23.1)	242(24.5)	
Consultation with an eye specialist due to screen-related eye issues?	Yes	224(44.3)	110(21.7)	172(34)	>0.001
	No	497(58.1)	206(24.1)	153(17.9)	
Eye symptoms interfering with daily activities	No impact	224(75.9)	45(15.3)	26(8.8)	>0.001
	Mild impact	382(54.7)	176(25.2)	140(20.1)	
	Moderate impact	99(32.8)	81(26.8)	122(40.4)	
	Severe impact	16(23.9)	14(20.9)	37(55.2)	

**Table 7:** Binary logistic regression analysis of variables with a *p* < 0.2 for the development of CVS.

Variables	*B*	S. E.	Wald	df	Sig.	Adjusted odds ratio (95% CI)
Age	−0.017	0.052	0.109	1	0.741	0.983 (0.888–1.088)
Sex (male)	−0.559	0.123	20.797	1	0.000	0.572 (0.45–0.727)
Study level			14.606	6	0.024	
1st year	0.269	0.404	0.444	1	0.505	1.309 (0.593–2.88)
2nd year	0.077	0.363	0.045	1	0.832	1.080 (0.53–2.2)
3rd year	0.163	0.330	0.244	1	0.621	1.177(0.62–2.3)
4th year	−0.355	0.287	1.530	1	0.216	0.701(0.4–1.23)
5th year	0.264	0.274	0.930	1	0.335	1.303(0.76–2.2)
6th year	−0.344	0.240	2.051	1	0.152	0.709(0.44–1.13)
Place of living (rural)	0.183	0.191	0.918	1	0.338	1.201(0.826–1.748)
Glasses			6.492	2	0.039	
Yes, but vision is not completely corrected	−0.415	0.171	5.905	1	0.015	0.660(0.47–0.92)
Yes, and vision is completely corrected	−0.189	0.182	1.069	1	0.301	0.828(0.579–1.2)
Eye_condition(yes)	−0.749	0.198	14.262	1	0.000	0.473(0.32–0.7)
Hour_per_day			11.199	4	0.024	
Less than 2 h	−1.702	0.781	4.753	1	0.029	0.182(0.03–0.84)
2–4 h	−0.576	0.236	5.976	1	0.015	0.562(0.35–0.89)
4–6 h	−0.317	0.163	3.801	1	0.051	0.728(0.53–1.00)
6–8 h	−0.141	0.155	0.829	1	0.363	0.869(0.64–1.18)
Tablet (yes)	−0.058	0.141	0.171	1	0.679	0.943(0.7–1.2)
Studying (yes)	0.143	0.146	0.969	1	0.325	1.154(0.87–1.5)
Breaks			1.528	3	0.676	
Every 30 min	−0.072	0.169	0.180	1	0.671	0.931(0.67–1.3)
Every 1 h	0.124	0.158	0.613	1	0.434	1.132(0.8–1.5)
Every 1–2 h	0.044	0.191	0.053	1	0.819	1.045(0.72–1.52)
Protection (yes)	0.103	0.144	0.508	1	0.476	1.108(0.84–1.5)
Screen brightness (yes)	0.188	0.134	1.962	1	0.161	1.207(0.93–1.6)
Screen filters (yes)	−0.189	0.132	2.033	1	0.154	0.828(0.64–1.1)
Lubricating eye drops (yes)	−1.002	0.185	29.277	1	0.000	0.367(0.26–0.53)
Previous ophthalmology (yes)	−0.373	0.149	6.264	1	0.012	0.688(0.51–0.92)
Impact_level			175.292	3	0.000	
No impact	−1.663	0.387	18.468	1	0.000	0.190(0.09–0.40)
Mild impact	−0.150	0.388	0.150	1	0.699	0.860(0.40–1.8)
Moderate impact	0.328	0.422	0.604	1	0.437	1.388(0.61–3.2)
Constant	4.379	1.386	9.989	1	0.002	79.795

Although our results showed that practices such as adjusting screen brightness, taking frequent regular breaks, using eye lubricants, and consulting an eye specialist were associated with lower CVS prevalence, these findings should be interpreted with caution. Given the cross-sectional design, causality cannot be established. It is also possible that students experiencing more severe symptoms were more likely to adopt these behaviors, which may reflect reverse causality rather than a true protective effect.

Findings from a large Egyptian study conducted among medical students highlighted the role of behavioral and environmental factors in reducing the burden of computer vision syndrome. Practices such as taking regular breaks, maintaining appropriate lighting conditions, using blue filters, and minimizing screen glare were associated with lower symptom frequency, supporting the importance of simple ergonomic modifications in improving visual comfort (35).

From a practical perspective, these findings can inform several interventions. Medical school administrators and curriculum planners may consider incorporating structured screen breaks into academic schedules and promoting ergonomically appropriate learning environments. Student health services could also play a role in early identification and management of CVS through screening and awareness initiatives. At the individual level, encouraging better screen use habits such as limiting continuous exposure, following the 20–20-20 rule, and optimizing viewing conditions may help reduce the symptom burden. These recommendations are consistent with previous research documenting the importance of behavioral and ergonomic modifications in reducing digital eye strain (35).

## Conclusion

Our study found a high prevalence of computer vision syndrome (CVS) among medical students at Cairo University (72.4%), highlighting the multifactorial nature of Computer Vision Syndrome (CVS) among students. Several factors were significantly associated with increased CVS prevalence and severity, including female sex (78.8%), lower academic levels, smoking (35.4%), pre-existing eye conditions (89.6%), incomplete vision correction, prolonged daily screen time, and the use of handheld devices such as tablets (78.4%). These findings align with previous research indicating higher CVS prevalence among female individuals and those with extended screen exposure. Importantly, certain protective behaviors such as adjusting screen brightness, taking regular breaks, consulting eye care professionals, and using eye lubricants were associated with reduced CVS symptoms. Our study provides detailed evidence from a large sample of medical students at one of the largest universities in Egypt. However, these findings should be interpreted with caution due to the cross-sectional nature of the study and the possibility of reverse causality.

### Limitations

One of the main limitations of our study is its cross-sectional design conducted at a single university. This limitation restricts the generalizability of the findings and precludes the establishment of a causal relationship or assessment of changes over time. The prevalence of CVS was reported based on subjective symptoms, which could lead to an inaccurate perception of symptom severity in the absence of objective ophthalmic examination. Although validated instruments such as the CVS-Q offer high sensitivity for screening symptoms, they may also overestimate the actual prevalence (35). In addition, the study did not include detailed screening for ocular conditions, psychological factors, and some environmental and ergonomic factors that may contribute to CVS.

### Strengths

In our study, we used a validated questionnaire (CVS-Q) to assess computer vision syndrome, enhancing the reliability of symptom evaluation. In addition, the study provides evidence from a large sample of medical students at one of the largest and most established medical schools in the Middle East and offers a comprehensive assessment of multiple risk factors associated with CVS. The use of the CVS-Q also allowed for standardized classification of symptom severity into distinct categories. Furthermore, the results were analyzed across different subgroups, enabling a more detailed understanding of the distribution and determinants of CVS among the study population. This study contributes to the growing body of literature on CVS and highlights the need for longitudinal and interventional research to better understand causal relationships and develop effective prevention strategies.

### Future research

Future studies should incorporate objective ophthalmological examinations for screening CVS, as well as a more detailed assessment of ocular conditions. In addition, psychological, environmental, and ergonomic factors that may contribute to CVS should be comprehensively evaluated. Future studies should assess the long-term effects of digital device use on ocular health and evaluate the need for targeted, evidence-informed interventions within academic settings. These may include promoting appropriate screen ergonomics, optimizing screen exposure patterns, and improving awareness of eye health practices among students. Future research should also examine the efficacy of various preventive strategies and investigate the role of emerging technologies in CVS mitigation.

Finally, research involving diverse populations can provide a more comprehensive understanding of CVS and help inform targeted interventions.

## Data Availability

The original contributions presented in the study are included in the article/supplementary material, further inquiries can be directed to the corresponding author.

## References

[1] Sheppard AL Wolffsohn JS. Digital eye strain: prevalence, measurement and amelioration. BMJ Open Ophthalmol. (2018) 3:e000146. doi: 10.1136/bmjophth-2018-000146, 29963645 PMC6020759

[2] Fatima F Ali A Sadiq SN Anwar A. To tackle symptoms of computer vision syndrome in era of COVID-19. Ophthalmol J. (2022) 7:140–3. doi: 10.5603/OJ.2022.0022

[3] León-Figueroa DA Barboza JJ Siddiq A Sah R Valladares-Garrido MJ Adhikari S . Prevalence of computer vision syndrome during the COVID-19 pandemic: a systematic review and meta-analysis. BMC Public Health. (2024) 24:640. doi: 10.1186/s12889-024-17636-5, 38424562 PMC10902934

[4] Gadain Hassan HA. Computer vision syndrome among medical students at the University of Khartoum, Sudan: prevalence and associated factors. Cureus. (2023) 15:e38762. doi: 10.7759/cureus.38762, 37303411 PMC10249515

[5] Yee NL Binti Muhammad Riduan Wong LFW Stewart MA Nor Haty Binti H Singh GKJ Bin Mohamad Anuar MF. Prevalence, knowledge and associated factors related to computer vision syndrome among undergraduate students. Florence Nightingale J Nurs. (2024) 32:118–25. doi: 10.5152/FNJN.2024.23037, 39545780 PMC11332467

[6] Al Dandan O Hassan A Al Shammari M Al Jawad M Alsaif HS Alarfaj K. Digital eye strain among radiologists: a survey-based cross-sectional study. Acad Radiol. (2021) 28:1142–8. doi: 10.1016/j.acra.2020.05.006, 32532637

[7] Mohan A Sen P Shah C Jain E Jain S. Prevalence and risk factor assessment of digital eye strain among children using online e-learning during the COVID-19 pandemic. Indian J Ophthalmol. (2021) 69:140–4. doi: 10.4103/ijo.IJO_2535_20, 33323599 PMC7926141

[8] Wang C Joltikov KA Kravets S Edward DP. Computer vision syndrome in undergraduate and medical students during the COVID-19 pandemic. Clin Ophthalmol. (2023) 17:1087–96. doi: 10.2147/OPTH.S405249, 37064959 PMC10103703

[9] Noreen K Ali K Aftab K Umar M. Computer vision syndrome (CVS) and its associated risk factors among undergraduate medical students in midst of COVID-19. Pak J Ophthalmol. (2020) 37:102–8. doi: 10.36351/pjo.v37i1.1124

[10] Abudawood GA Ashi HM Almarzouki NK. Computer vision syndrome among undergraduate medical students in king Abdulaziz university, Jeddah, Saudi Arabia. J Ophthalmol. (2020) 2020:1–7. doi: 10.1155/2020/2789376

[11] Iqbal M El-Massry A Elagouz M Elzembely H. Computer vision syndrome survey among the medical students in Sohag University hospital, Egypt. Ophthalmol Res Int J. (2018) 8:1–8. doi: 10.9734/OR/2018/38436

[12] 1. Raosoft Inc. Sample Size Calculator. Seattle, WA: Raosoft I. (2025). Available online at: http://www.raosoft.com/samplesize.htm (accessed May 15, 2025).

[13] Alfarhan H. Validation of DEQ-5 unidimensionality and differential item functioning of Saudi adult populations. Invest Opthalmol Vis Sci. (2026) 65:1–6. doi: 10.70706/iovs.95.15.125

[14] Artime Ríos E Sánchez Lasheras F Suárez Sánchez A Iglesias-Rodríguez F Seguí Crespo M. Prediction of computer vision syndrome in health personnel by means of genetic algorithms and binary regression trees. Sensors. (2019) 19:2800. doi: 10.3390/s19122800, 31234490 PMC6630344

[15] Larese Filon F Drusian A Ronchese F Negro C. Video display operator complaints: a 10-year follow-up of visual fatigue and refractive disorders. Int J Environ Res Public Health. (2019) 16:2501. doi: 10.3390/ijerph16142501, 31337021 PMC6678724

[16] Cantó-Sancho N Porru S Casati S Ronda E Seguí-Crespo M Carta A. Prevalence and risk factors of computer vision syndrome—assessed in office workers by a validated questionnaire. PeerJ. (2023) 11:e14937. doi: 10.7717/peerj.14937, 36890870 PMC9987297

[17] Rahman ZA Sanip S. Computer user: demographic and computer related factors that predispose user to get computer vision syndrome. Int J Business, Humanit Technol. (2011) 1:84–91.

[18] Ranasinghe P Wathurapatha WS Perera YS Lamabadusuriya DA Kulatunga S Jayawardana N . Computer vision syndrome among computer office workers in a developing country: an evaluation of prevalence and risk factors. BMC Res Notes. (2016) 9:150. doi: 10.1186/s13104-016-1962-1, 26956624 PMC4784392

[19] Toomingas A Hagberg M Heiden M Richter H Westergren KE Tornqvist EW. Risk factors, incidence and persistence of symptoms from the eyes among professional computer users. Work A J Prev Assess Rehabil. (2014) 47:291–301. doi: 10.3233/WOR-131778 24284674

[20] Portello JK Rosenfield M Chu CA. Blink rate, incomplete blinks and computer vision syndrome. Optom Vis Sci. (2013) 90:482–7. doi: 10.1097/OPX.0b013e31828f09a7, 23538437

[21] Sullivan DA Rocha EM Aragona P Clayton JA Ding J Golebiowski B . TFOS DEWS II sex, gender, and hormones report. Ocul Surf. (2017) 15:284–333. doi: 10.1016/j.jtos.2017.04.001, 28736336

[22] Saad NE Ahmed MM Yousef FF Almehelmy EM. Computer vision syndrome and associated factors among students of Faculty of Medicine, Cairo University. Med J Cairo Univ. (2019) 87:4877–81. doi: 10.21608/mjcu.2019.85230

[23] Sengo DB da Deolin Bernardo Pica A Dos Santos d’AlvaII B Mate LM Mazuze AN Caballero P . Computer vision syndrome and associated factors in university students and teachers in Nampula, Mozambique. BMC Ophthalmol. (2023) 23:508. doi: 10.1186/s12886-023-03253-0, 38093208 PMC10720210

[24] Pavel IA Bogdanici CM Donica VC Anton N Savu B Chiriac CP . Computer vision syndrome: an ophthalmic pathology of the modern era. Medicina. (2023) 59:412. doi: 10.3390/medicina59020412, 36837613 PMC9961559

[25] Abdolalizadeh HA. Computer Vision Syndrome. London: IntechOpen (2026). doi: 10.5772/intechopen.87234

[26] Abed Alah M Abdeen S Selim N AlDahnaim L. Computer vision syndrome among students during remote learning periods: harnessing digital solutions for clear vision. Front. Public Health. 11:1273886. doi: 10.3389/fpubh.2023.1273886 PMC10666185 38026296

[27] Matsumoto Y Dogru M Goto E Sasaki Y Inoue H Saito I . Alterations of the tear film and ocular surface health in chronic smokers. Eye. (2008) 22:961–8. doi: 10.1038/eye.2008.78, 18425058

[28] Steigerwalt R D Jr Laurora G Incandela L Cesarone M R Belcaro G V MTDS. Computer Vision Syndrome Among Students During Remote Learning Periods: Harnessing Digital Solutions for Clear Vision. (2026). 10.3389/fpubh.2023.1273886 PMC10666185 38026296

[29] Gammoh Y. Digital eye strain and its risk factors among a university student population in Jordan: a cross-sectional study. Cureus. (2021) 13:e13575. doi: 10.7759/cureus.13575, 33815983 PMC8007199

[30] Mutti DO Zadnik K. Is computer use a risk factor for myopia? J Am Optom Assoc. (1996) 67:521–30. 8888885

[31] Altalhi A Khayyat W Khojah O Alsalmi M Almarzouki H. Computer vision syndrome among health sciences students in Saudi Arabia: prevalence and risk factors. Cureus. (2020) 12:e7060. doi: 10.7759/cureus.7060, 32226662 PMC7089631

[32] Han L Zhang H Xiang Z Shang J Anjani S Song Y . Desktop lighting for comfortable use of a computer screen in a dark environment. Work. (2021) 68:S209–21. doi: 10.3233/WOR-208018 33337420 PMC7902945

[33] Reddy SC Low C Lim Y Low L Mardina F Nursaleha M. Computer vision syndrome: a study of knowledge and practices in university students. Nepal J Ophthalmol. (2013) 5:161–8. doi: 10.3126/nepjoph.v5i2.8707, 24172549

[34] Wangsan K Upaphong P Assavanopakun P Sapbamrer R Sirikul W Kitro A . Self-reported computer vision syndrome among Thai university students in virtual classrooms during the COVID-19 pandemic: prevalence and associated factors. Int J Environ Res Public Health. (2022) 19:3999. doi: 10.3390/ijerph19073996, 35409679 PMC8997620

[35] Iqbal M Elzembely H Soliman A. Computer vision syndrome prevalence and ocular sequelae among medical students: a university-wide study on a marginalized visual security issue. Open Ophthalmol J. (2021) 15:15–23. doi: 10.2174/1874364102115010156

